# Constitutively Active Lck Kinase in T Cells Drives Antigen Receptor Signal Transduction

**DOI:** 10.1016/j.immuni.2010.05.011

**Published:** 2010-06-25

**Authors:** Konstantina Nika, Cristiana Soldani, Mogjiborahman Salek, Wolfgang Paster, Adrian Gray, Ruth Etzensperger, Lars Fugger, Paolo Polzella, Vincenzo Cerundolo, Omer Dushek, Thomas Höfer, Antonella Viola, Oreste Acuto

**Affiliations:** 1 T Cell Signaling Laboratory, Sir William Dunn School of Pathology, University of Oxford, Oxford OX1 3RE, UK; 2 Istituto Clinico Humanitas IRCCS, I-20089 Rozzano (Milano), Italy; 3 MRC Human Immunology Unit, Weatherall Institute of Molecular Medicine, University of Oxford OX3 9DU, UK; 4 Tumour Immunology Group, Weatherall Institute of Molecular Medicine, University of Oxford OX3 9DU, UK; 5 Sir William Dunn School of Pathology and Center for Mathematical Biology, University of Oxford, Oxford OX1 3RE, UK; 6 Research Group Modeling of Biological Systems, German Cancer Research Center, 69120 Heidelberg, Germany; 7 Department of Translational Medicine, University of Milan and Istituto Clinico Humanitas IRCCS, I-20089 Milano, Italy

**Keywords:** MOLIMMUNO, CELLIMMUNO, SIGNALING

## Abstract

T cell antigen receptor (TCR) and coreceptor ligation is thought to initiate signal transduction by inducing activation of the kinase Lck. Here we showed that catalytically active Lck was present in unstimulated naive T cells and thymocytes and was readily detectable in these cells in lymphoid organs. In naive T cells up to ∼40% of total Lck was constitutively activated, part of which was also phosphorylated on the C-terminal inhibitory site. Formation of activated Lck was independent of TCR and coreceptors but required Lck catalytic activity and its maintenance relied on monitoring by the HSP90-CDC37 chaperone complex to avoid degradation. The amount of activated Lck did not change after TCR and coreceptor engagement; however it determined the extent of TCR-ζ phosphorylation. Our findings suggest a dynamic regulation of Lck activity that can be promptly utilized to initiate T cell activation and have implications for signaling by other immune receptors.

## Highlights

► Active Lck is present in unstimulated T cells ► Lck activity is not increased after TCR ligation ► Active Lck can be phosphorylated on both activating and inhibitory sites ► Lck activation is independent of TCR and coreceptors

## Introduction

Src family kinases (SFKs) couple many cell-surface receptors to the intracellular signaling machinery to regulate proliferation, differentiation, survival, and adhesion dynamics (Bromann et al., 2004; Parsons and Parsons, 2004). This SFK's multitasking ability probably reflects versatility in substrate targeting and cell context-specific regulation. T cell receptor (TCR) signaling upon peptide-MHC (pMHC) binding requires phosphorylation of immunoreceptor tyrosine-based activation motifs (ITAMs) within the CD3 complex by the SFK Lck (Palacios and Weiss, 2004). Phosphorylated ITAMs recruit and activate the Syk protein tyrosine kinase (PTK) ZAP-70 to set in motion pathways that orchestrate T cell proliferation and differentiation (Acuto et al., 2008; Au-Yeung et al., 2009). Conventional views of T cell activation suggest that pMHC binding activates Lck. However, a mechanistic basis for this model, implying a direct link between TCR and Lck, is lacking. Because Lck is stably associated to the coreceptors CD4 and CD8 (Rudd et al., 1988; Veillette et al., 1988), other models have suggested that binding to MHC activates Lck by trans-autophosphorylation. Indeed, coreceptor oligomerization by antibody crosslinking induces increased Lck tyrosine phosphorylation and activity (Holdorf et al., 2002; Veillette et al., 1989). However, structural and membrane biophysical studies do not support the existence of stable and/or abundant MHC dimers or oligomers (Baker and Wiley, 2001; Cherry et al., 1998), and there is no direct experimental evidence that MHC binding activates Lck. Moreover, coreceptors are partially dispensable for T cell development and activation (Van Laethem et al., 2007). Thus, the lack of strong experimental support for these models leaves unanswered the central mechanism that initiates TCR-mediated adaptive immunity.

The activity of SFKs is largely controlled by the equilibrium between phosphorylation and dephosphorylation at a C-terminal inhibitory tyrosine and an activating tyrosine in the catalytic domain (Boggon and Eck, 2004; Yamaguchi and Hendrickson, 1996). When phosphorylated by the C-terminal Src-kinase (Csk), the C-terminal tyrosine binds intramolecularly to the SH2 domain (Xu et al., 1999), which, together with the SH3 domain interacting with a PPII α-helix packed against the kinase domain, locks SFKs into an autoinhibited closed form (Boggon and Eck, 2004). The SH3-SH2 “clamp” must be released to initiate activation. Dephosphorylation of the C-terminal tyrosine by membrane-bound protein tyrosine phosphatases (e.g., PTPα, CD45) helps relax the closed form (Hermiston et al., 2003; Su et al., 1999). Kinase unwinding may be also favored by binding of the SH3-SH2 module to substrate(s) or regulatory partner(s) (Boggon and Eck, 2004). However, catalytic activation entails tyrosine phosphorylation at the activation loop to precisely position the ATP phosphates, the amino acids involved in catalysis, and the substrate (Boggon and Eck, 2004). Ligand-induced collision of receptor-associated SFKs may lead to trans-autophosphorylation of the activation loop. Alternatively, SFKs may be activated by receptor tyrosine kinases (RTKs) after binding to them (Bromann et al., 2004). However, the exact mechanism of these processes that usually induce modest SFK activation is unclear (discussed in Bromann et al., 2004), and thus far it is unknown whether SFK activation is exclusively instructed by external stimuli.

We previously reported biochemical evidence for the presence of activated Lck (pY394-Lck) in transformed human T cell lines and normal T cells prior to TCR stimulation (Irles et al., 2003). More recently, Alexander and coworkers (McNeill et al., 2007) also detected activated SFKs in isolated mouse primary T cells and thymocytes. However, the lack of evidence that activated Lck is expressed in unstimulated cells in vivo and lack of quantification of activated Lck precluded reaching any conclusion on the relevance and consequences of these observations. Here, we showed that relatively high amounts of pY394-Lck (and pY418-Fyn) are expressed in T cells and thymocytes in isolation and in situ in lymphoid tissues. B cells also do express preactivated SFKs, although at lower amounts than T cells (data not shown). Approximately half of pY394-Lck was a new form phosphorylated on both the activation and inhibitory site, which has kinase activity equivalent to Lck phosphorylated only on Y394. Basal amounts of pY394-Lck were regulated dynamically by trans-autophosphorylation and maintained by the HSP90-CDC37 chaperone complex. Preactivated Lck was sufficient for inducing activation because TCR ligation did not lead to further increase of pY394-Lck or its kinase activity. However, reduction of basal pY394-Lck critically affected TCR signaling initiation and sensitivity. By revealing a special regulatory mechanism to maintain activated SFKs in T cells, our data prompt a revision of the current models of TCR signal transduction.

## Results

### Activated SFKs Are Expressed in Unstimulated T Cells and Thymocytes In Vitro and In Vivo

**Figure 1:**
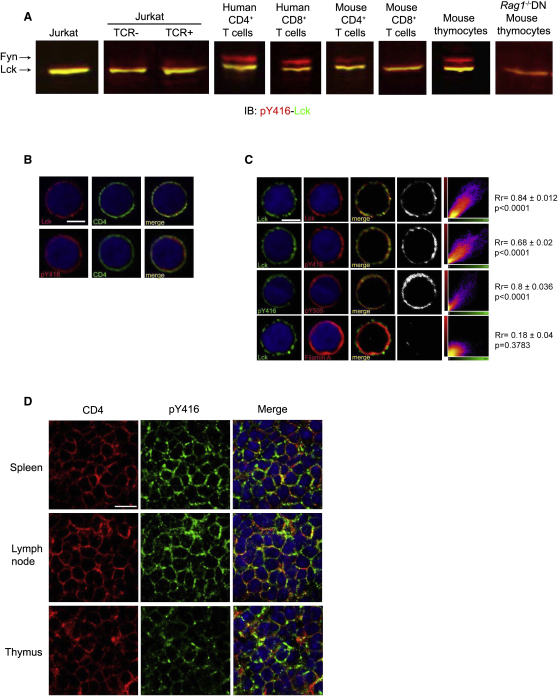
Activated SFKs in T Cells and Thymocytes (A) Dual fluorescence immunoblot (IB) for Lck (green) and pY416 (red) in Jurkat cells, TCR-, CD4-, and CD8-deficient 31.13 (TCR-) and TCR-reconstituted 31.13 (TCR+) Jurkat cells, human and mouse CD4^+^ and CD8^+^ T cells, and mouse thymocytes from normal and *Rag*1^−/−^ mice. Merged (yellow) denotes expression of pY394-Lck (see Figure S1C). The ∼59 kDa species is mostly pY418-Fyn. (B) Confocal IF of human CD4^+^ T cells stained for CD4 and either for Lck or active SFKs (pY416). Nuclei were counterstained with Hoechst 33258 (blue). The scale bar represents 3.5 μm. (C) Confocal IF for Lck forms in human CD4^+^ T cells. Double staining with two different Abs to Lck, a rabbit Ab (red) and a mouse mAb (green) (first row), was used as the positive control for colocalization. Double staining with a rabbit Ab to Lck (green) and a mAb to Filamin A (red) (bottom row) was the negative control (lack of colocalization). The presence of pY394-Lck was identified by double staining with Lck mAb (green) and pY416 rabbit Ab (red) (second row). For pY394 and pY505 colocalization (third row), we used anti-pY416 rabbit Ab (green) and anti-pY505 mAb (red). The degree of colocalization was determined with Imaris colocalization software. Corresponding pictures were merged (third column) and all pixels colocalized were represented as white spots (fourth column). Each colocalized pixel was plotted on a scatter diagram for producing correlation plots with colocalizing pixels falling around the diagonal line (right-hand column). Data from at least 50 cells for each condition were used for calculating the Pearson's correlation coefficient (R_r_) as mean ± SE p values (two-tailed) determined with Student's t test. The scale bar represents 3.5 μm. (D) Confocal IF images of a mouse spleen, LN, and thymus. Tissue sections (10 μm) were stained with biotin-labeled anti-mouse CD4 (L3T4) (red) and anti-pY416 (rabbit) (green). The scale bar represents 10 μm. Images are representative of three independent experiments.

To investigate the regulation of Lck in T cells, we used pY416 Ab that identifies the activated form of several SFKs, including Lck (Figure S1A) and pY505 Ab that binds to the phosphorylated inhibitory site of Lck. Specificity controls excluded undesirable cross-reactivities of these reagents (see “Specificity of Anti-pY416 and Anti-pY505-Lck Abs in Immunoblot and Immunofluorescence” in Supplemental Results and Figures S1B–S1E available online). The pY416 Ab readily detected activated Lck (pY394-Lck) in cell lysates of unstimulated Jurkat cells, human and mouse T cells and thymocytes (Figure 1A). The identity of the activated Lck was confirmed by anti-Lck immunoprecipitation followed by anti-pY416 immunoblotting (Figure S1F). The 59 kDa species reacting with anti-pY416, more prominent in human CD4^+^ T cells and mouse thymocytes, was identified as being activated Fyn (pY418-Fyn) (Figure S1F). Cell culture (e.g., serum-free or serum-enriched medium) or lysis conditions (e.g., ice-cold lysis or at 100°C in Laemmli sample buffer) did not appear to alter the amounts of activated SFKs (not shown). In contrast, cultured HeLa cells showed barely detectable activated Fyn (Figure S1F). These results are indicative of differential regulation mechanisms of SFK activity within distinct cell types.

Immunofluorescence (IF) analysis of human CD4^+^ T cells with Lck or pY416 Abs intensely stained the cell surface decorated for CD4 (Figure 1B), consistent with Lck and activated SFKs being present mostly at the plasma membrane. To directly identify active Lck in intact cells, we carried out IF colocalization studies with Lck and pY416 Abs (Figure 1C). Control double staining with two distinct Lck Abs gave, as expected, high colocalization (Pearson's correlation coefficient, Rr. close to 1.0), whereas Lck and Filamin A showed poor colocalization as predicted for two spatially distant proteins. Staining with pY416 and Lck Abs showed strong colocalization indicating, in accordance with the immunoblot data, the presence of activated Lck. Separate Lck (green) or pY416 (red) pixels were presumably due, in part, to nonactivated Lck and activated Fyn, respectively. Collectively, these data indicated that cultured unstimulated T cells and thymocytes expressed activated forms of SFKs, confirming and extending previous in vitro studies (Irles et al., 2003; McNeill et al., 2007). It was crucial to determine whether activated SFKs were also present in intact lymphoid tissues. To this purpose, we carried out in situ staining with anti-pY416 in the mouse spleen, lymph node (LN), and thymus. Anti-pY416 stained the majority of CD4^+^ T cells and thymocytes (Figure 1D). Similar data were obtained for CD8^+^ T cells (not shown). Moreover, similar T cell staining with anti-pY416 was observed when tissues were obtained from paraformaldehyde (PFA)-perfused animals (not shown), excluding changes during manipulation. Pretreatment with alkaline phosphatase (AP) abolished anti-pY416 reactivity (Figure S1E), providing a control for the specificity of the in situ staining. To quantitatively link activated SFK detected in situ and in vitro, we directly compared staining with anti-pY416 in situ in spleen and in cells isolated from part of the same organ (Supplemental Experimental Procedures). Parallel staining with anti-pY416 revealed similar fluorescence intensity (Figure S1G), excluding that the amount of activated SFKs substantially changed during cell isolation. Taken together, these data provided evidence that normal T cells and thymocytes do express in vivo activated SFKs, including Lck and Fyn.

### Quantification of Activated Lck in T Cells

**Figure 2:**
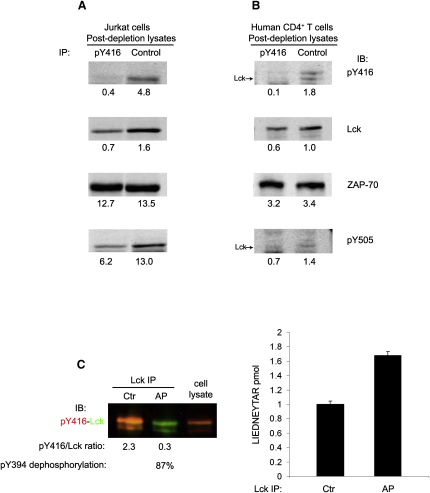
Quantification of pY394-Lck and pY505-Lck in T Cells (A and B) Denatured lysates from (A) Jurkat cells or (B) human CD4^+^ T cells were split into two halves and subjected to three rounds of immunoprecipitations (IP) with pY416 Ab (pY416) or rabbit IgG (Control), respectively. Lysates were analyzed by immunoblotting (IB) for residual pY394-Lck, Lck, and pY505-Lck. The loading control was ZAP-70. IBs were scanned for fluorescence and relevant bands were quantified. Numbers below each panel correspond to the actual fluorescence signals. (C) Lck isolated from Jurkat cells with the 3A5 mAb was split in two and incubated with (AP) or without (Ctr) alkaline phosphatase for 30 min at 37°C. The fraction of pY394 dephosphorylated was determined by immunoblotting (left panel). Lck from 10^7^ cells (twice for each condition) was in-gel digested with trypsin, after spiking with 1 pmol of [L-C^13^N^15^] IEDNEYTAR. Each sample was analyzed twice by MS. Histogram represents mean picomoles ± SE, n = 4 of endogenous LIEDNEYTAR in AP-treated and untreated samples. Details of AQUA and MS are available in the Supplemental Information.

The apparent abundance of activated Lck in T cells prompted us to estimate its relative proportion over total Lck. To this end, we initially carried out immunodepletions of pY394-Lck from Jurkat lysates by using anti-pY416 and measured the loss of Lck by anti-Lck quantitative immunoblotting (Figure 2A). We used denatured lysates to rule out coimmunoprecipitation of potentially occurring oligomers of Lck forms. Control experiments revealed negligible levels (∼1%–2%) of inactive Lck (Y394-Lck) carried over by anti-pY416 depletions (Figure S2A and S2B). In Jurkat cells, depleting >90% of pY394-Lck, resulted in a >50% reduction of total Lck (56% ± SD 2.1% [n = 3]), indicating that more than half of Lck was phosphorylated on Y394 (Figure 2A). Similar results were obtained with nondenatured cell lysates (data not shown).

To validate the immunodepletion data in Jurkat cells by an independent approach, we estimated pY394-Lck over total Lck by mass spectrometry (MS) by using Absolute Quantification (AQUA) (Gerber et al., 2003). Because of the partial digestion of the tryptic peptide containing phosphorylated Y394, an indirect but reliable procedure was preferred (see Supplemental Experimental Procedures). Thus, a heavy isotope-labeled peptide, [L-C^13^N^15^]IEDNEYTAR, corresponding to the Lck tryptic fragment containing nonphosphorylated Y394 was used for quantifying Lck in samples untreated or treated with AP, which dephosphorylates pY394 (Figure 2C). The difference between these two measures provided an estimate of pY394-Lck. The experiment in Figure 2C showed that AP treatment (87% dephosphorylation of pY394) increased the amount of Y394-Lck from ∼1.0 to ∼1.7 pmoles. After correction for 100% dephosphorylation, Lck amounted to ∼1.9 pmoles, which translated into 0.9 pmoles of pY394-Lck, 47% (0.9/1.9) of total Lck, in good agreement with the immunodepletion data. Anti-pY416 immunodepletion of human naive CD4^+^ T cell lysates (one of which is illustrated in Figure 2B) indicated that ∼37% ± 8.5% (n = 3) of Lck was activated, with some variability between donors.

To corroborate these data, we compared by immunoblotting pY394-Lck/Lck ratio in Jurkat cells (used as a reference for the proportion of active Lck over the total Lck, e.g., 56%) to the pY394-Lck/Lck ratios in human naive CD4^+^ T cells from four additional donors (Figure S2C). CD4^+^ T cell content of activated Lck was ∼65% of that found in Jurkat cells. This translates into 36% ± 4.3% (n = 4) of activated Lck in normal T cells, in agreement with the immunodepletion experiments. Comprehensively, these data indicated that Jurkat and normal T cells can express activated Lck in a high proportion, yet they do not become spontaneously activated.

### Activated Lck Exists in Two Major Forms

**Figure 3:**
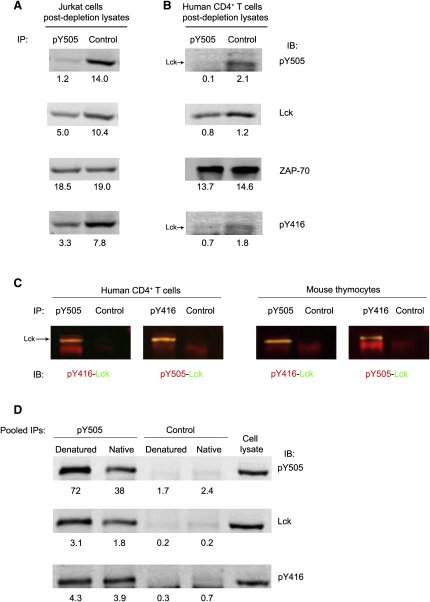
A Proportion of Lck Is Phosphorylated at Both Y394 and Y505 (A and B) Denatured lysates from (A) Jurkat cells or (B) human CD4^+^ T cells were treated as in Figures 2A and 2B but immunoprecipitated with anti-pY505 rabbit Ab (pY505) or rabbit IgG (Control). Postdepletion lysates were probed for residual pY505-Lck, Lck, and pY394-Lck. The loading control was ZAP-70. Signal intensity was detected as in Figures 2A and 2B. (C) Denatured lysates of human CD4^+^ T cells (left panels) or total mouse thymocytes (right panels) were immunoprecipitated with anti-pY505 or anti-pY416 or rabbit IgG (Control) and immunoblotted with anti-pY416 (red) or anti-pY505 (red), respectively, and with anti-Lck (green). Merged images (yellow) reveal pY394-Lck containing pY505 and vice versa. (D) Native or denatured Jurkat lysates were immunoprecipitated three times with anti-pY505 or rabbit IgG. Immunoprecipitates were pooled, immunoblotted for pY505, Lck, and pY394-Lck (with anti-pY416), and quantified. These experiments were performed three times with similar results.

pY505-Lck should represent inactive Lck. To estimate its relative proportion with respect to active Lck, we carried out depletion experiments by using pY505 Ab (Figures 3A and 3B). We found that 52% ± 2.6% (n = 3) and 35% ± 4.6% (n = 3) of total Lck was phosphorylated at pY505 in Jurkat and naive human CD4^+^ T cells, respectively. Quantitative immunoblot experiments comparing pY505/Lck ratios (Figure S2D) indicated that human naive CD4^+^ T cells contained ∼56% of the pY505 expressed in Jurkat cells, giving 30% ± 8.2% (n = 4) of pY505-Lck, in good agreement with the immunodepletion data. However, we noticed that complete removal of pY394-Lck in Jurkat cells led also to a loss of ∼50% of pY505-Lck (Figure 2A, bottom panel) and depletion of pY505-Lck also removed ∼50% of pY394-Lck (Figure 3A, bottom panel). Similar to Jurkat cells, in human CD4^+^ T cells approximately half of pY394 or pY505 was removed after depletion with anti-pY505 and anti-pY416, respectively (Figures 2B and 3B). Because Lck was denatured, it was unlikely that active and inactive forms were coimmunoprecipitated and in such a close proportion with the two Abs. Thus, a likely explanation was that a fraction of pY394-Lck was also phosphorylated at the inhibitory site. This form, pY394-pY505-Lck (hereafter referred as to “double” phosphorylated, DPho-Lck), was directly demonstrated to exist not only in T cells but also in thymocytes by showing that Lck isolated with anti-pY416 from these cells contained pY505 and vice versa (Figure 3C). Expression of DPho-Lck was also confirmed in intact T cells by the strong colocalization of anti-pY505 and anti-pY416 staining (Figure 1C, third row). To our knowledge, DPho-Lck has not been noted before, and it represents a sizable fraction of total Lck (∼20%–30%, Table 1 and see “Estimation of the Relative Proportions of the Four Lck Forms” in Supplemental Results for calculations).

DPho-Lck may be locked in a closed conformation similar to the inactive form of Lck with the phosphorylated C-terminal inhibitory site stably interacting with SH2 domain (Xu et al., 1999) and therefore sterically hindered (Kabouridis, 2003). If however DPho-Lck is in an open conformation, the phosphorylated C-terminal might react with the pY505 Ab. To test this idea, we carried out immunodepletions with anti-pY505 of intact and denatured lysates of Jurkat cells and probed them by immunoblotting for pY394, Lck, and pY505 (Figure 3D). Under native conditions, anti-pY505 reacted with Lck that was also phosphorylated at pY394. However, not all pY505-Lck could be depleted (data not shown). After denaturation, anti-pY505 quantitatively immunodepleted pY505-Lck and approximately twice as much pY505 and Lck was found, although the amount of pY394 remained essentially unchanged. These data are consistent with DPho-Lck being in an open conformation with pY505 disengaged from the SH2 domain and thus readily accessible to the antibody. In contrast, in inactive-closed Lck (Y394-pY505) the phosphorylated C-terminal is not accessible (see Figure S3 for a scheme of Ab accessibility) and becomes exposed only after protein denaturation. The data obtained by the immunodepletion experiments shown in Figures 2 and 3 indicated that Lck exists in four major forms according to the phosphorylation at the activation and inhibitory sites and provided an estimation of the proportion of each of them (Table 1 and see “Estimation of the Relative Proportions of the Four Lck Forms” in Supplemental Results).

### pY394-Lck and DPho-Lck Have Similar In Vitro Kinase Activity

**Figure 4:**
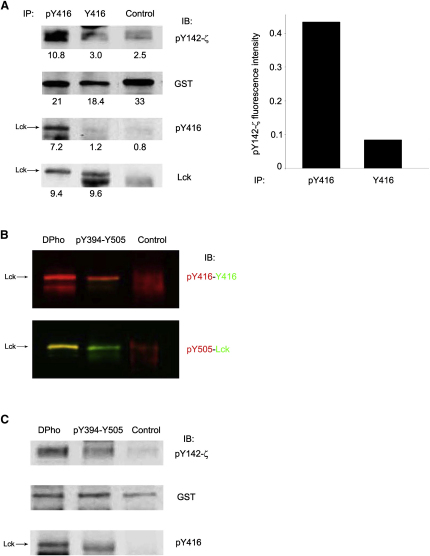
Catalytic Activity of Dpho-Lck (A) Lck activity is mostly associated with pY394-Lck. Jurkat lysates were immunoprecipitated with anti-pY416 or anti-Y416 or with control IgG (Control) and subjected to in vitro kinase assay on rCD3-ζ-GST. The reaction mixture was immunoblotted for pY142-ζ, GST, pY394 (pY416), and Lck and quantified by near-infrared fluorescence (numbers below each panel). Overlapping Lck and pY416 fluorescence (not shown) provide positive identification of pY394-Lck. Kinase activity (right panel) was expressed as pY142-ζ signal subtracted for IgG control and normalized for rCD3-ζ-GST. (B and C) Dpho- and pY394/Y505-Lck have similar kinase activity. As shown in (B), DPho (pY394-pY505) and pY394 (pY394-Y505) were individually enriched by immunoprecipitation (see Results). An aliquot was subjected to dual fluorescence immunoblotting with anti-pY416 (red) and anti-Y416 (green) or anti-pY505 (red) and anti-Lck (green). The control was rabbit IgG. In (C), Dpho- and pY394-Lck from (B) and IgG control immunoprecipitations were assayed for kinase activity on rCD3-ζ-GST as in (A) and immunoblotted for pY142-ζ, GST and pY394 (pY416). Identification of pY394-Lck was as in (A). A representative experiment of three giving similar results is shown.

DPho-Lck appeared to be in an open conformation; thus, we examined whether it was catalytically active. We used Jurkat cells because they contained negligible amounts of Fyn or other SFKs. We first employed anti-pY416 and anti-Y416 to isolate pY394-Lck and Y394-Lck pools, respectively, and compared their kinase activity on recombinant CD3-ζ-GST [rCD3-ζ]). As expected (Boggon and Eck, 2004), the vast majority of kinase activity was associated with pY394-Lck (Figure 4A). We next separated the two Lck forms containing pY394 and compared their activity on rCD3-ζ (Figures 4B and 4C). DPho-Lck was isolated with anti-pY505 and contained mostly pY394-Lck and only traces of Y394-Lck (Figure 4B). To enrich for pY394-Lck, we sequentially depleted DPho-Lck with anti-pY505 and then inactive and primed Lck (Y394-pY505- and Y394-Y505-Lck) with anti-Y416. The remaining pY394-Lck was then immunoprecipitated with anti-Lck and was strongly enriched for pY394/Y505-Lck (compared with the strong signal of anti-pY416 and weak signal of anti-pY505 and anti-Y416 in Figure 4B). We found that DPho-Lck and pY394-Y505-Lck displayed comparable kinase activity (Figure 4C, lanes 1 and 2) and correlated with similar amounts of pY394-Lck in both fractions (Figure 4C). Thus, activated Lck is found in two forms that appear to have similar capacity to phosphorylate TCR ITAMs and are present in nonstimulated T cells in a high proportion.

### Regulation of Basally Activated Lck in T Cells

In view of our data, we examined which mechanisms might be responsible for generating and maintaining basally active Lck. One hypothesis is that activated SFKs might be controlled by TCR and/or coreceptor interactions with self-pMHC. However, we detected high amounts of activated Lck in CD4 and CD8-negative (double negative [DN]) thymocytes from Rag-deficient mice that lack expression of pre-TCR (Figure 1A) and in DN thymocytes from normal mice (not shown). Moreover, comparable amounts of pY394-Lck were found in cultured Jurkat cells irrespective of TCR, CD4, and CD8 expression (Figure 1A). Activated Lck and Fyn persisted in blood-derived human naive T cells (depleted of MHC class II-expressing cells) (Figure 1A) after several hours of diluted cultures, conditions in which the TCR or CD4 are unlikely to be engaged. Furthermore, CD4 IPs from Jurkat cells did not show any preferential binding of active Lck to the coreceptor (Figure S4). Thus, expression of activated SFKs did not appear to depend on TCR or CD4 and CD8 coreceptor expression and/or their contact with pMHC.

**Figure 5:**
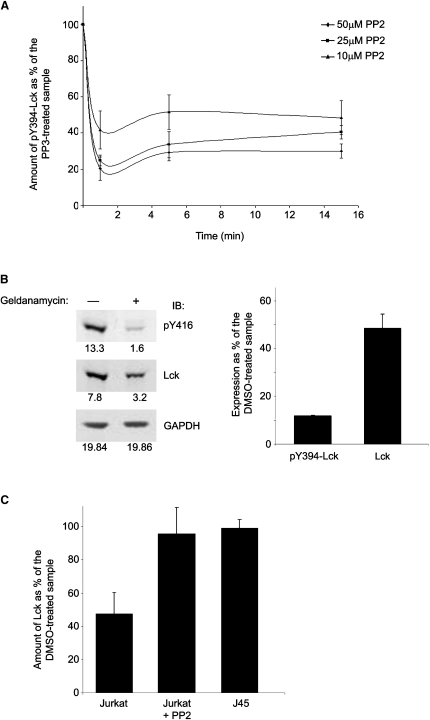
Basal Amounts of Active Lck Are Regulated by Its Own Kinase Activity and Binding to the HsP90-CDC37 Chaperone Complex (A) Jurkat cells were treated with different concentrations of PP2 or its inactive analog PP3. The amount of pY394-Lck was quantitated by anti-pY416 immunoblotting at the indicated times. Error bars represent standard deviation from three independent experiments. (B) Jurkat cells were treated with 5 μM Geldanamycin, or DMSO as control, for 3 hr at 37°C. Cell lysates were probed with anti-pY416 and anti-Lck, and GAPDH was used as a loading control. The histogram on the right represents data collected from three independent experiments. (C) Jurkat cells preincubated with 50 μM PP2 or its inactive analog PP3, or J45 cells, were treated with 5 μM Geldanamycin or DMSO, as in (B). The amount of Lck was determined by anti-Lck immunoblotting and protein loading by GAPDH (not shown). Error bars represent standard deviation from three independent experiments

SFK activation may be attributable to trans-autophosphorylation at the conserved activation loop tyrosine (Palacios and Weiss, 2004). To test this hypothesis, we treated Jurkat cells with various concentrations of PP2, a very potent and specific inhibitor of SFKs, or PP3, an inactive PP2 analog, as a control. Cellular amounts of activated Lck decayed with an exceptionally fast half-life (∼30 s) at all doses of PP2 tested (Figure 5A), reaching different basal amounts. These data suggested that Lck kinase activity is critical to maintain its own basal activated state that is tightly and very dynamically controlled by a potent PTPase, plausibly CD45 (McNeill et al., 2007).

Previous work has established that the stability and expression of constitutively active Lck mutants require association with the HSP90-CDC37 chaperone complex (Giannini and Bijlmakers, 2004; Prince and Matts, 2004). We therefore investigated whether this was also the case for basally activated Lck by treating cells with geldanamycin, a specific inhibitor of HSP90 binding to client proteins (Giannini and Bijlmakers, 2004). Treatment of Jurkat cells with geldanamycin for 3 hr resulted in a dramatic decrease of pY394-Lck (∼90%) concomitant with a ∼50% loss of the total Lck (Figure 5B), coincident with ∼50% of Lck being in the active form in Jurkat cells (Table 1). In contrast, little or no degradation of Lck, in response to geldanamycin, was observed in J45 cells, which express markedly reduced amounts of CD45 (Koretzky et al., 1991), (Figure 5C). In this cell line, the majority of Lck is phosphorylated on Y505 thus existing in the closed-inactive conformation and containing very small amounts of activated Lck compared to Jurkat (data not shown). Similarly, little loss of Lck after geldanamycin treatment was observed in Jurkat cells treated with PP2 (Figure 5C), with the latter resulting in >80% reduction on the basal level of pY394-Lck (data not shown). These data suggest that constant monitoring by the HSP90-CDC37 chaperone complex is essential for preserving the basal amounts of activated Lck by means of protecting it from proteolytic degradation.

### TCR Signaling Does Not Increase but Requires Basally Activated Lck

**Figure 6:**
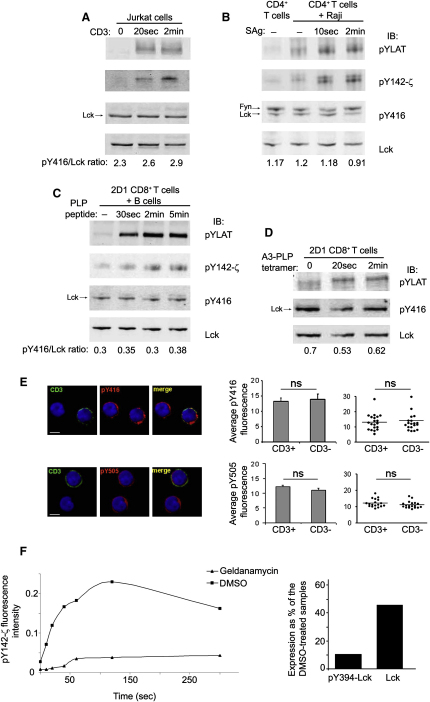
TCR Ligation Does Not Increase pY394-Lck or Lck Kinase Activity (A) Jurkat cells stimulated for the indicated times with anti-CD3. An aliquot was probed with anti-pY, anti-pY142-ζ, and anti-pY416. Lck (bottom panel) was a loading control. (B) Human CD4^+^ T cells were added for the indicated times to unpulsed or sAg-pulsed Raji B cells. Cell activation was detected by pYLAT and pY142-ζ as in (A). Lysates were also probed for pY394-Lck (with anti-pY416) and Lck. The ∼59 kDa species is mostly Fyn. (C) 2D1 TCR-tg CD8^+^ T cells were stimulated with either unpulsed or PLP peptide-pulsed LPS-activated B cells from MHC class I HLA-A3 tg mice. Cell activation and pY394-Lck changes were monitored as in (B). (D) 2D1 TCR-tg CD8^+^ T cells were stimulated for the indicated times with A3-PLP-MHC class-I tetramers. Cell activation and pY394 changes were detected as in (C). Each of these experiments was performed at least twice. (E) Confocal IF of purified human CD4^+^ T cells left untreated or stimulated with anti-CD3 for 2 min at 37°C. Cells were fixed, mixed at a ratio 1:1, and stained with anti-pY416 (top panels) or anti-pY505 (bottom panels), revealed by anti-rabbit Alexa594 (red), and with anti-mouse Alexa488 (green) to detect the presence of anti-CD3. The histograms represent the average anti-pY416 or anti-pY505 fluorescence intensity measurements, in 15 cells, from single-focal planes chosen at random ± SE (13.26 ± 1.4 and 13.59 ± 1.7 n = 15 ROI. p < 0.05) in unstimulated (CD3^−^) and stimulated (CD3^+^) cells respectively. The dot plots on the right represent the distribution of fluorescence intensity/cell for the same set of cells. The scale bar represents 5 μm. (F) Jurkat cells treated for 3 hr with 5 μM Geldanamycin, or DMSO as control, were stimulated for the indicated times with anti-CD3. Cell lysates were probed with anti-pY142-ζ and the intensity of the bands was quantitated by near-infrared fluorescence. Anti-pY142-ζ values, normalized for protein loading (by GAPDH immunoblotting), were plotted against stimulation time so that induction of ζ chain phosphorylation could be designated. The histogram shows the reduction on pY394 and total Lck resulting from geldanamycin treatment. One representative experiment out of three is shown.

The relatively high amount of activated Lck in T cells raised the question as to whether TCR-induced activation required further increase in Lck activity. We employed different stimulation settings of Jurkat cells as well as human and mouse T cells, by using anti-CD3, *Staphylococcus* enterotoxin superantigen (sAg)-MHC class II and peptide-MHC class I, respectively. The latter two forms of stimulation involved coreceptor recruitment near the TCR. Activation of Jurkat cells with saturating anti-CD3 strongly increased tyrosine phosphorylation of CD3-ζ (pY142-ζ) and LAT. However, no reproducible trend was seen toward augmentation of the pY394-Lck/Lck ratio (∼ ±10%) (Figure 6A), and only small variations around the mean were observed in many experiments. Consistently, no appreciable change of the in vitro kinase activity was recorded within the pY394, total Lck, or the Y394-Lck fractions (Figure S5). Also, we did not observe reproducible changes in pY505 amounts at these time points (data not shown). Human CD4^+^ T cells were stimulated with sAgs-pre-pulsed Raji B cells, conditions that activated most cells (>80%). Whereas sAg stimulation induced CD3-ζ and LAT tyrosine phosphorylation at early time points, no augmentation in pY394-Lck content was detected (Figure 6B). Moreover, 2D1 TCR-tg mouse CD8^+^ T cells (Friese et al., 2008) stimulated with activated B cells loaded with a maximal dose of agonist antigen peptide presented by class I MHC induced CD3-ζ and LAT tyrosine phosphorylation without substantial change in the pY394-Lck content (Figure 6C). Finally, pY394-Lck did not increase when 2D1 TCR-tg CD8^+^ T cells were stimulated with a saturating dose of a strong agonist tetramer A3-PLP-MHC class-I (Figure 6D). In addition, quantitative confocal IF analysis of anti-CD3 stimulated human CD4^+^ T cells revealed no changes in the fluorescence intensity of pY416 or pY505 staining as compared to unstimulated cells (Figure 6E). Together, these data suggested that the substantial proportion of activated Lck present in resting T cells was sufficient to initiate TCR signaling and that no further activated Lck was needed.

In view of these findings, we examined the significance of the existence of high amounts of activated Lck for TCR signaling. We used geldanamycin as a tool to selectively obtain reduced amounts of basally activated Lck (Figures 5B and 6F) and then stimulated Jurkat cells with anti-CD3 for different times, monitoring the onset and amplitude of CD3-ζ phosphorylation by anti-pY142-ζ immunoblotting. Geldanamycin treatment had no impact on the protein amount of LAT, ZAP-70, CD3-ζ chain, or GAPDH (not shown). An example of these experiments is shown in Figure 6F. Cells expressing only ∼10% of activated Lck, with only half reduction of total Lck (right panel), showed severely compromised ζ chain phosphorylation (Figure 6F). Furthermore, although in control cells induction of pY142-ζ was recorded as early as 10 s of stimulation, cells with reduced basal pY394-Lck demonstrated no recordable ζ phosphorylation at this early time point (graph, left). These data suggest that high amounts of preactivated Lck are essential for the potency and rapidity of TCR signal propagation.

## Discussion

The mechanism regulating the ability of Lck to induce ITAM phosphorylation, one of the earliest signaling events detected after TCR ligation, has remained elusive. Although it is commonly believed that Lck is activated upon TCR and/or coreceptor engagement, direct proof for this model is lacking. In this study, we provided strong evidence that resting T cells expressed relatively high amounts of Lck constitutively phosphorylated on the activation loop. This pool, which contained most Lck kinase activity presumably geared up for but uncoupled from ITAM phosphorylation, appeared to remain unchanged upon TCR and coreceptor ligation, suggesting that it suffices to initiate T cell activation. These data are consistent with recent FRET analysis that detected no Lck conformational changes upon TCR ligation (Paster et al., 2009). Some studies, but not others (Filipp et al., 2003; Holdorf et al., 2002; and the present work), have reported small increases over background in Lck tyrosine phosphorylation and/or kinase activity upon TCR stimulation (Burkhardt et al., 1994). Differences in stimulatory conditions, cell solubilization, quantitative monitoring of Lck phosphorylation, and activity changes with a specific substrate may explain this discrepancy. In our study, multiple experiments using different stimulatory settings consistently revealed no changes in the amount of basally active Lck. Quantitative SILAC-based MS of anti-CD3-stimulated Jurkat cells also found no substantial change above background in Lck pY394 phosphorylation (data not shown). We cannot rule out that undetectable increases in activated Lck may take place in the proximity of the TCR, which may be relevant for ITAM phosphorylation. However, our data indicate that such a mechanism would require a pool of pre-existing activated Lck the abundance of which determines the level of TCR-induced ITAM phosphorylation. Imaging studies detected pY394-Lck accumulated at the immunological synapse minutes after T cell-APC interaction, presumably because of coreceptor engagement, which was suggested to reflect stimulation-induced activation of Lck (Campi et al., 2005; Holdorf et al., 2002). However, because cells were not imaged prior to APC contact, it was not documented whether activated Lck was present before stimulation, as we show in our study. We detected activated SFKs in T cells in vitro and in situ in all lymphoid compartments examined, suggesting a cell context-specific regulation of SFK activity in the T cell lineage. In situ and in vitro data suggest that unstimulated B cells also express basally activated SFKs, although at lower amounts than T cells (data not shown).

Activated Lck did not appear to depend on “tonic stimuli” from the TCR and/or coreceptors and was preserved in unstimulated cultured lymphocytes. This is consistent with data suggesting that T cells deprived in vivo of self-MHC contact for prolonged periods of time show intact TCR-induced activation (Fischer et al., 2007). If TCR and coreceptors do not seem to control the amount of activated Lck, what then regulates its generation and maintenance? The high and constitutive PTP activity of CD45 ensures dephosphorylation at the autoinhibitory site over Csk action (Palacios and Weiss, 2004) and generates primed Lck, which we found to be a sizable fraction of the total Lck pool. Primed Lck is likely to be the precursor that generates activated Lck by trans-autophosphorylation at pY394. Inhibition of SFK activity by PP2 resulted in a very rapid decay of pY394 amounts, implying that Lck activity is dynamically kept in check by a potent PTP, most likely CD45 (Irles et al., 2003; McNeill et al., 2007); yet it has a sufficient kinetic advantage over the PTP to form relatively high amounts of active Lck. One possibility is that localization of Lck in membrane domains from which most CD45 is excluded (Irles et al., 2003) favors the formation of active Lck. However, additional mechanisms employing regulatory partners, such as Tsad (Marti et al., 2006) and Unc119 (Gorska et al., 2004), could contribute to facilitating opening of the kinase (Boggon and Eck, 2004) and/or collision-induced trans-autophosphorylation.

We also showed that constitutively active wild-type Lck requires binding to HSP90-CDC37 in order to prevent its degradation. However, this was not the case for the primed and closed-inactive forms of Lck. Thus, the amount of basally active Lck is maintained by at least two regulatory mechanisms, a trans-autophosphorylation exhibiting a sufficient kinetic advantage over the action of a PTP and a balance between HSP90-CDC37-dependent stabilization and protein degradation.

The naturally occurring DPho-Lck in T cells was open, as revealed by anti-pY505 binding, and it had kinase activity comparable to pY394-Y505-Lck. We therefore assume that the SH2-SH3 module in DPho-Lck is free to bind to substrates and that its kinase domain is in a fully active conformation. DPho-Lck may be the consequence of abundant generation of pY394-Lck in T cells, and at present, we favor the hypothesis that it derives from pY394-Y505-Lck phosphorylated by Csk. Indeed, if inactive-closed Lck was the direct precursor of DPho-Lck, the latter might have remained in a closed conformation (Moarefi et al., 1997). The presence of DPho-Lck may be the result of membrane compartmentalization and/or of Lck interacting partners that help preserve phosphorylation of unbound pY505 in spite of the strong CD45 activity. Although future studies will address these questions, a consequence of our findings is that pY505-Lck in T cells does not solely represent inactive Lck.

In agreement with constitutively activated Lck being used for ITAM phosphorylation, we showed that reduction of this form correlated with decreased TCR-induced ITAM phosphorylation. We estimated that unstimulated normal T cells can express up to ∼10^4^ activated Lck molecules, most of which are plasma membrane bound. This is a high value considering Lck *k*_cat_ for CD3-ζ ITAMs (1–2 s^-1^) and the high copy number of CD3-ζ ITAMs (1.8 × 10^5^) for 3 × 10^4^ TCRs. Using a simple model for enzymatic reactions within the membrane, on the basis of these and other established values, we estimate that when few TCRs are engaged, those levels of activated Lck allow phosphorylation of 3-6 ITAMs on a single TCR within 3–6 s, which is in agreement with the observed fast kinetics of TCR-triggered signaling (Huse et al., 2007). If smaller numbers of activated Lck are considered in our model (e.g., 10^2^–10^3^), then phosphorylation of ITAMs requires tens of seconds. Consistently, we found that the onset of CD3-ζ phosphorylation in response to TCR ligation was delayed in cells containing strongly reduced (∼90%) amounts of activated Lck. Preliminary data of Jurkat cells treated for different times with geldanamycin showed a very close correlation between the amount of basally activated Lck and the amplitude of CD3-ζ ITAMs phosphorylation (data not shown). Thus, constitutively activated Lck appears to be required to shorten the time lapse from ligand binding to ITAM phosphorylation and to confer high sensitivity to TCR-induced signaling.

A central question emerging from our findings is how activated Lck is prevented from extensively phosphorylating the ITAMs (and other receptor tails) in unstimulated cells although it is “licenced” to do so within seconds after TCR ligation. It has been suggested that TCR intracellular tails interact with acidic membrane lipids and are exposed to Lck phosphorylation and ZAP-70 binding only after TCR ligation (Sigalov et al., 2006; Xu et al., 2008). Moreover, there is evidence for ligand-induced TCR-α chain conformational (Beddoe et al., 2009) and TCR-CD3 quaternary changes (Kim et al., 2009), which may lead to intracellular conformational changes (Gil et al., 2002; Xu et al., 2008) and to Lck access to the ITAMs. In addition to that, in T cells SFKs (and coreceptors) are dynamically confined into liquid-ordered lipid microdomains from which nonengaged TCRs may be excluded (Douglass and Vale, 2005; He and Marguet, 2008; Shimada et al., 2005), thus limiting TCR contact with activated SFKs. TCR ligation may induce conformational changes and/or changes in the lipid environment around the TCR (Shimada et al., 2005; Zech et al., 2009), which, together with concomitant engagement of coreceptor carrying activated Lck, facilitate TCR ITAM phosphorylation.

In view of the present findings, we propose a “standby” model for SFKs in T cells, in which regulated amounts of preactivated Lck, kept under strict control by CD45, are necessary and sufficient to ensure TCR-induced ITAM phosphorylation. This model bypasses the need for coreceptor dimerization or oligomerisation to activate Lck and may explain TCR signaling in the absence of coreceptors. One interesting corollary of the “standby” model is that other immune receptors that signal via highly diverse intracellular tails and use SFKs would not require individually specialized mechanisms to activate SFKs but would exploit a common preactivated pool, perhaps by using signal transduction rules similar to antigen receptors. Finally, our data reveal the versatility of SFKs regulation, which may have been specifically adapted for the purpose of lymphocyte activation.

## Experimental Procedures

### Reagents

Reagents, antibodies, and cells used are described in the Supplemental Information.

### Immunoprecipitation and Immunoblotting

Cultured cells were rapidly centrifuged and the pellet was immediately lysed with ice-cold lysis buffer, 20 mM Tris-HCl (pH 7.5), 150 mM NaCl, 1% n-Dodecyl-β-D-maltoside (LM), 1 mM Na_3_VO_4_, and 1 mM phenylmethylsulphonyl fluoride. n-Dodecyl-β-D-maltoside (LM) quantitatively extracts SFKs from detergent-resistant membranes (DRMs). Clarified lysates were incubated with the indicated Abs for 2 hr at 4°C and subsequently for 1 hr with protein A/G-agarose beads (Santa Cruz Biotechnology). Immune complexes were washed in lysis buffer and boiled in Laemmli sample buffer. For immunodepletion of denatured Lck, cells were lysed in lysis buffer containing 0.4% LM. Clarified lysates were treated 5 min at 100°C in the presence of 1% v/v SDS, diluted 10-fold by adding 1% LM-containing buffer to quench SDS, and subjected to three rounds of immunoprecipitation with the indicated Abs or with normal rabbit or mouse IgG. Lysates and immunoprecipitates were separated by SDS-PAGE and immunoblots quantified by near-infrared fluorescence with the Odyssey imaging system (LI-COR Biosciences).

### Kinase Assay

Lck immunoprecipitates were isolated with the indicated Abs, washed three times in kinase buffer (20 mM Tris-HCl [pH 7.5], 10 mM MgCl_2_, 10 mM MnCl_2_, and 1 mM ATP), and incubated for 20 min at 37°C in 20 μl of kinase buffer together with 100 ng of GST-CD3-ζ. The reaction was stopped by adding 4× Laemmli sample buffer at 100°C, and Lck activity was quantified by near-infrared fluorescence with anti-pY142-ζ immunoblotting.

### Confocal Immunofluorescence

Immunofluorescence staining and confocal microscopy on isolated cells and tissues is described in the Supplemental Information.

### Cell Stimulation and Drug Treatment

A total of 10^7^ purified human CD4^+^ T cells were incubated at a 3:1 ratio with Raji B cells, either unpulsed or pulsed with a mixture of 0.5 μg/ml SEA, 0.5 μg/ml SEB and 0.5 μg/ml SEC for the indicated times at 37°C. A total of 3 × 10^6^ Jurkat cells were stimulated at 37°C with anti-CD3 (UCHT1) at 10 μg/ml. A total of 10^7^ TCR transgenic 2D1 CD8 T cells were stimulated at 37°C at a ratio 1:1 with LPS-activated HLA-A3 mouse-derived B cells prepulsed with 50 μM PLP 45-53 peptide. 2D1 CD8 T cells were activated by soluble HLA-A3-PLP 45-53 tetramer (Friese et al., 2008) at concentrations that gave saturating staining in flow cytometry.

Cells were incubated with 5 μM 17- (Allylamino)-17-demethoxy geldanamycin (17-AAG) for 3 hr at 37°C and either lysed immediately or stimulated with anti-CD3 as described above. PP2 was added at increasing concentrations (10–50 μM) at 37°C and the cells were lysed at different time points.

## Figures and Tables

**Table 1:** T Cells Express Four Major Functional Forms of Lck

	Relative Proportion of Lck Forms in Unstimulated Cells^a^			
	Closed-Inactive^b^	Primed	pY394-Active	DPho-Active
Human CD4^+^ T cells	14%	48%	17%	21%
Jurkat cells	23%	25%	23%	29%

a The calculations of the relative proportions of Lck forms are explained in “Estimation of the Relative Proportions of the Four Lck Forms” in the Supplemental Results.

b Column headings are described as follows: closed-inactive, Lck retains the SH2 intramolecular binding and locks the kinase into a catalytically compromised conformation; primed Lck, phosphorylated neither at Y505 nor Y394. The SH3 domain-linker binding may still be preserved; pY394-active, Lck kinase domain assumes an optimal conformation for catalysis; and DPho-Active, The Dpho-Lck contains both pY394 and pY505 and assumes an open conformation like pY394-active.

## Supplemental Information

Document S1. Supplemental Experimental Procedures, Supplemental Results, and Five Figures
