# Supplementary material for: Constitutively Active Lck Kinase in T Cells Drives Antigen Receptor Signal Transduction
Source: Immunity. 2010 Jun 25;32(6):766–77. doi: 10.1016/j.immuni.2010.05.011 (PMC2996607; doi:10.1016/j.immuni.2010.05.011)
Supplement: Document S1. Supplemental Experimental Procedures, Supplemental Results, and Five Figures [file mmc1.pdf]

Immunity, Volume *32*

Supplemental Information

Constitutively Active Lck Kinase in T Cells Drives Antigen Receptor Signal Transduction

Konstantina Nika, Cristiana Soldani, Mogjiborhaman Salek, Wolfgang Paster, Adrian Gray, Ruth Etzensperger, Lars Fugger, Paolo Polzella, Vincenzo Cerundolo, Omer Dushek, Thomas Höfer, Antonella Viola, and Oreste Acuto

Supplemental Experimental Procedures

Reagents used in this work

Anti-Lck, anti-pY505-Lck, anti-pY416-Src rabbit Abs and anti-Y416-Src (7G9) mouse mAb were from Cell Signaling Technology; anti-Lck (3A5), anti-Fyn (64A406) mouse mAbs, anti-CD4 (YNB46.1.8) rat mAb, anti-GST rabbit Ab and normal rabbit IgG were from Santa Cruz Biotechnology; anti-Lck (Ab8075) mouse mAb was from Abcam; anti-v-Src (Ab-1), anti-pY505-Lck mAb (clone 4), anti-pY142-ζ (K25-407.69), anti-human CD3ε (UCHT1) and PE-conjugated form, anti-human CD4 (SK3), PE-anti-human CD4 (L120), PE-anti-human CD45 (HI30), FITC-anti-human CD8 (G42-8) mouse mAbs, anti-mouse CD19 (1D3) rat mAbs, Alexa488-anti-mouse CD3ε (145-2C11) hamster mAbs and purified mouse IgG2b were purchased from BD Pharmigen; anti-CD3 (UCHT1) was from Serotec; biotin-labelled anti-mouse CD4 (L3T4) and anti-mouse CD8 (53-6.7) rat mAbs were from eBioscience; anti-LAT rabbit Ab, anti-ZAP-70 (2F3.2), anti-pY (4G10) mouse mAbs were from Upstate Biotechnology; anti-Filamin (FLMN01) mouse mAb was from Thermo Scientific. Secondary Abs for infrared fluorescence immunoblot were IRDye800CW goat anti-mouse IgG and IRDye680 goat anti-rabbit IgG (LI-COR Biosciences). Secondary Ab for chemiluminescence was sheep anti-rabbit HRP-coupled from GE Healthcare. Indirect immunofluorescence secondary Abs were Alexa488-anti-mouse IgG, Alexa488-anti-rabbit IgG, Alexa594-anti-rabbit IgG, Alexa594-anti-mouse IgG, Alexa 647-anti-rabbit IgG, Alexa594-anti-rat IgG and Alexa594-streptavidin (Molecular Probes).

PP2, PP3 and 17- (Allylamino)-17-demethoxy geldanamycin (Calbiochem). Alkaline phosphatase and laurylmaltoside (Sigma). SEA, SEB and SEC (Toxin Technology). Recombinant CD3-ζ-GST (Abnova).

MHC class I HLA-A3 tetramers were previously described (Friese et al., 2008). Lck-Y394F and Y505F mutant cDNAs cloned into pEF-neo expression vector (a gift of T. Mustelin, Amgen).

Cells

The Jurkat T cells and the variants Lck-deficient JCaM.1 and CD45-deficient J45 (a gift of A. Weiss, UCSF), TCRβ chain-deficient 31.13 and TCRβ-reconstituted, and Raji B cell lymphoma were grown in RPMI-1640-10% FCS. HeLa cells were grown in DMEM-10% FCS. Human naïve $CD4^+$ T cells and human $CD8^+$ T cells (>95 % pure) were isolated by negative selection from whole blood of healthy donors (National Blood Service, Bristol, UK) using Dynal isolation kit (Invitrogen). Cells were routinely maintained in culture for 6-12 h before lysis. Cells were isolated from normal or *Rag*-$1^{-/-}$ thymi and spleen or LN from C57BL/6 mice. C57BL/6 CD4 T cells (95%) and B cells (> 98%) or CD8 T cells from TCR transgenic (tg) 2D1 mice (Friese et al., 2008) were isolated by negative selection using MACs (Miltenyi Biotec) kit. Mouse cells were cultured for 5 h before lysis. In control experiments primary human and mouse T cells were lysed immediately after isolation.

Immunofluorescence of isolated cells and tissues

For immunofluorescence, human $CD4^+$ T cells were isolated by negative selection using RosetteSep kit (StemCell Technologies) and cultured at 37°C in RPMI-10% FCS before use. Cells were fixed in 4% paraformaldehyde (PFA), adhered to poly-L-lysine-coated microscope slides and permeabilized with PBS/Tween 0.1%. After incubation with PBS/1%BSA for 15 min, cells were stained for 1h at room temperature (RT) with the indicated primary Abs. Fluorochrome-conjugated secondary Abs for double staining were added for 1h. Nuclei were counterstained with Hoechst 33258 (1 μg/ml). Negative controls used secondary antibodies alone. Images were acquired by a confocal microscope Fluoview FV1000 (Olympus) with an oil immersion objective (60x 1.4 NA Plan-Apochromat; Olympus) using laser excitation at 405, 488 and 543nm. Images were processed using Adobe Photoshop 9.0.2. For colocalization analysis, images were acquired by a confocal microscope as above except that a

pinhole of 0.9 Airy Units. Images were acquired at a digital size of 1024x1024 pixels and a scan speed of 40 ms/pixel was used. Differential interference contrast was also used. Colocalization was measured on a single confocal section corresponding to the major diameter membrane focus, using the 'Colocalization' module of Imaris 5.0.1, 64-bit version (Bitplane AG; for details see (Marvizon et al., 2007). Corresponding pictures were merged and all pixels co-localized were identified. The brightness of each co-localized pixel in the optical section shown was plotted on the x and y axes of a scatter diagram to give correlation plots. Pixels clustering near the origin of the axes indicate lack of co-localisation whereas co-localizing pixels fall around the diagonal line of the plot. All experiments were performed using different pairs of fluorochrome-conjugated secondary Abs. Experimental procedures with animals were performed in accordance with the Italian and European regulations governing the care and treatment of laboratory animals (Permit No. 94/2000A). For *in situ* staining, inguinal LNs, spleen and thymus from C57BL/6 mice were frozen in OCT. 10 μm cryostat tissue sections were fixed in formalin for 10 min, permeabilized with PBS/Tween 0.1% and blocked with PBS/1%BSA for 15min. Staining was carried out at RT with the indicated primary (2 h) and secondary Abs (1 h). In a few experiments, staining was performed on sections from LNs and spleens immediately PFA-fixed after isolation or from PFA-perfused mice. Slides were mounted with ProLong (Molecular Probes). To directly compare levels of activated SFKs in intact tissue and cell suspension, a mouse spleen was cut into two pieces and one half was frozen in OCT. The other half was processed to obtain a debris-free single cell suspension that was then centrifuged and the pellet snap-frozen. Ten μm slices of the cell pellet and the intact spleen and placed onto the same slide. Staining was performed as described above.

Quantitative near-infrared immunofluorescence

Membranes were scanned with the LI-COR Odyssey infrared imaging system using the manufacturer's settings for "membranes" (resolution: 169μm, quality: medium, focus offset: 0.0, channels 700nm and 800nm). Channel(s) were chosen according to the secondary Ab used: 800nm for IRDye® 800CW (green) and 700nm IRDye® 680 (red). Accurate coincident green and red fluorescence was controlled using a software function that showed fluorescence as peaks. The limits of quantification were assessed by systematic assays in which Lck from Jurkat cell lysates was detected in quadruplicates over a range of two and half orders of magnitude and found to be reliable.

Quantification was found to be reliable in the 700 nm channel (red) and 800 nm channel (green) at arbitrary intensities > 0.4, which was the limit for the red channel. Values of 0.1 or below were essentially background. After correction for gel loading, the error between samples was found to be <10% of the average.

Mass spectrometry

rLck (Upstate Biotechnology) or Lck isolated from Jurkat cells was separated by SDS-page and stained by Colloidal coomassie blue. The gel slice corresponding to Lck was excised and fragmented into small pieces, placed in a 0.5 ml Eppendorf tube and digested as described elsewhere (Shevchenko et al., 2006). In brief, the protein was reduced by 10mM dithioerythritol for 30 min at RT and alkylated by 55mM iodoacetamide for 30 min at RT in the dark. Overnight digestion at 37ºC was carried with 0.6µg trypsin (Proteomics Grade, Sigma) in 25mM ammonium bicarbonate. The resulting peptides were acidified with 1% trifluoroacetic acid (TFA) prior to loading onto a StageTip (Rappsilber et al., 2003) or Oasis® µElution plate (Oasis® HLB µElution plate, 30µm, Waters). Remaining peptides were extracted by incubating of the gel pieces in 50µl 1% TFA for 15 min. The new extract was loaded onto the previous StageTip or plate. Peptides bound to the solid phase were washed with 1% TFA and then eluted in 50 µl buffer B (50% acetonitrile, 0.1% formic acid) and lyophilized in a SpeedVac concentrator (Concentrator 5301, Eppendorf AG, Hamburg). Lyophilized peptides were re-suspended in 0.1% formic acid and subsequently analyzed by nanoLC-MS/MS. An LTQ-Orbitrap mass spectrometer (ThermoElectron, Bremen, Germany) (Dunn School of Pathology Proteomics facility) was coupled online to nano-LC (Ultimate, Dionex, USA). To prepare an analytical column, C_{18} phase (ReproSil-Pur C18-AQ 3µm; Dr. Maisch GmbH, Ammerbuch-Entringen, Germany) was packed into a spray emitter (75 µm ID, 8 µm opening, 70 mm length; New Objectives, USA). Mobile phase A consisted of water, 5% acetonitrile and 0.5 % acetic acid and mobile phase B of acetonitrile and 0.5% acetic acid. The five most intense peaks of the MS scan were selected in the ion trap for MS^2, (Normal scan, filling 5e5 ions, maximum injection time 500ms for MS scan, 10^4 ions for MS^2 and 200ms maximum injection time with multistage activation enabled, dynamic exclusion for 60 seconds). Raw-files were processed using DTAsupercharge v.1.19 (http://msquant.sourceforge.net/). The generated peak lists were searched against the IPI human database using Mascot 2.2 with the following parameters: monoisotopic masses, 10ppm on MS and 0.5 Da on MS/MS, ESI TRAP

parameters, full tryptic specificity, cysteine carbamidomethylated as fixed modification, oxidation on methionine, phosphorylation on Serine, Threonine, Tyrosine, protein N-acetylation and deamidation on glutamine and Asparagine as variable modifications, three missed cleavage sites allowed.

AQUA and estimation of Lck molecules/cell

Heavy isotope-labelled peptide corresponding to the Lck tryptic fragment containing Y394, [L-$C^{13}N^{15}$] IEDNEYTAR, was obtained from Sigma. A second isotope-labelled peptide, LIEDNEYT[A-C^{13}]REG[A-C^{13}]K (a generous gift of Dr. Wolf-Dieter Lehmann, DKFZ, Heidelberg, Germany) was also used which contains, in addition, a trypsin cleavage site, thus serving as an internal control for complete digestion and to duplicate absolute quantification. Once cleaved after in-gel digestion (see below) it could be distinguished for its mass from [L-$C^{13}N^{15}$] IEDNEYTAR. The lyophilized synthetic peptides were resuspended in 0.1% formic acid, the sequence verified by mass spectrometry and the elution times were determined under the same LC conditions to be used in AQUA experiments. For AQUA quantification (Gerber et al., 2003) MS data were analyzed by extracting total ion current of individual peptides and the area under the curve by using Xcalibur 2.0.6 Qual Browser. The identity of each peptide was validated first by its MS/MS spectra and further confirmed by i) accurate m/z value and ii) the presence of co-eluting doublet (light and heavy forms of the same peptides). If, for a given peptide, MS/MS spectra were missing, accurate m/z and co-elution criteria were used in combination with the previously determined LC elution times. Experimental conditions were optimized for complete digestion with known amounts of rLck isolated by SDS-PAGE and in-gel digested, after adding 1pmol of [L-$C^{13}N^{15}$] IEDNEYTAR peptide. Under the digestion condition used, we found an excellent agreement between the amount of rLck and the amount of LIEDNEYTAR (which is not phosphorylated in rLck, see Figure S1) detected by AQUA. To quantify the content of total Lck and the fraction phosphorylated on Y394, we immunoprecipitated Lck from a denatured lysates of 130 x 10^6 Jurkat cells. Under the conditions used, ~ 50 % of total Lck was immunoprecipitated as verified by quantitative immunoblot. One half was treated with alkaline phosphatase (AP) for 30 min at 37ºC (~ 90 % dephosphorylation, Figure 2C) and the other half was mock treated. Lck equivalent to ~ 10^7 cells/lane in duplicate was separated by SDS-PAGE, stained by colloidal coomassie blue and gel slices corresponding to Lck were added to trypsin digestion buffer containing 1pmol each of L-C13N15]IEDNEYTAR and LIEDNEYT[A- C13]REG[A- C13]K (the concentration in water of these

peptides was verified by amino acid composition quantitative analysis). No trace of the latter peptide was found uncleaved as assessed by MS analysis, suggesting complete digestion of the endogenous LIEDNEYTAR. After MS analysis, the ratio of heavy (labelled synthetic peptides) over light (endogenous equivalent Lck tryptic peptide) was calculated for the AP-treated and untreated Lck. This allowed determination of the absolute amounts of total Lck (AP-treated, corrected for 100% dephosphorylation of pY394) and Lck non-phosphorylated of Y394 in Jurkat cells. The difference between these two measures yielded the amount of pY394-Lck in Jurkat cells. This indirect method was preferred over the use of a heavy isotope-labelled pY394-containing tryptic peptide as we experienced inconsistent results in preliminary assays using auto-phosphorylated rLck presumably due to incomplete cleavage of LIEDNEpYTAR peptide. This may result from the formation of a salt bridge between the phosphate and the +3 arginine (Woods et al., 2005). In contrast, quantitative cleavage was observed for the endogenous LIEDNEYTAR. We found that 10^7 Jurkat cells contained ~1.9 pmoles Lck (Figure 2C), which corresponds to ~ 125,000 Lck molecules/cell. We confirmed this data by quantitative anti-Lck (3A5 Ab) immunoblot in triplicate using near-infrared fluorescence. Briefly,10 ng rLck were added to a lysate of 150,000 Lck-deficient Jurkat, JCaM.1 cells, and compared to a Jurkat lysate of 150,000 cells (not shown). Ten ng of rLck (1.07 x 10^{11} molecules) gave a fluorescence signal of 15.44 SD ± 0.55, n=3 and 150,000 Jurkat cells gave 2.83 ± 0.22, n=3. This corresponds to ~ 130,000 Lck molecules/cell, in excellent agrement with the AQUA measurements. Jurkat cells were found by quantitative immunoblot to contain ~ 5 fold more Lck/cell than human naive $CD4^+$ T cells (not shown), which corresponds to 25,000 Lck/cell.

Supplemental Results

Specificity of Anti-pY416 and Anti-pY505-Lck Abs in Immunoblot and Immunofluorescence

Mass spectrometry analysis of rLck identified tryptic peptides LIEDNEYTAR (the non-phosphorylated positive regulatory site within the activation loop) (m/z, 612.300), SVLEDFFTATEGQYQPQP (the non-phosphorylated auto-inhibitory site) (m/z, 1028.981) and its tyrosine phosphorylated form, SVLEDFFTATEGQpYQPQP (m/z, 1068.961) (Figure S1B) but did not detect LIEDNEpYTAR (the phosphorylated positive regulatory site). This peptide (m/z, 655.791) (Figure S1B) was detectable only

after *in vitro* auto-phosphorylation of rLck and in Lck isolated from Jurkat cells (Figure S1B) indicating that rLck is not phosphorylated at the activation loop. Consistently, anti-pY416 Ab (pY416) did not react in immunoblot with rLck but reacted only with auto-phosphorylated rLck or Lck from Jurkat cells (Figure S1C, top, left panels). Anti-pY505-Lck Ab (pY505) reacted with rLck but this reactivity was lost when the latter was dephosphorylated by alkaline phosphatase (AP) (Figure S1C, top, right panels). These experiments demonstrated that anti-pY416 Ab did not cross-react with its non-phosphorylated determinant on Lck (Y394) or with the phosphorylated Y505 inhibitory site (pY505). To complete these controls, Lck-Y505F and Lck-Y394F mutants were transiently expressed in Lck-defective JCam.1 Jurkat cells, which resulted in high levels of phosphorylated pY394 and pY505, respectively. Figure S1C, bottom panels shows that the former was not recognized by anti-pY505, thus confirming its specificity for the pY505 site. Collectively, these data indicated that anti-pY416 and anti-pY505-Lck polyclonal Abs used in our studies were strictly specific for pY394- and pY505-containing determinants, respectively, and were therefore reliable reagents for qualitative and quantitative assessment of the presence of phosphorylation sites defining functional states of Lck in cell-extracts. To assess the specificity of anti-pY416 and anti-pY505-Lck in immunofluorescence (IF) staining settings, isolated human naïve $CD4^+$ T cells were left untreated or treated in culture at 37ºC for 30 min with 100μM PP2 and then processed for IF staining using anti-pY416 and anti-pY505. Figure S1D shows that after this treatment IF with both Abs was drastically reduced while anti-Lck staining remained. The same figure shows also that anti-pY416, anti-pY505 and anti-Lck staining disappeared in Lck-deficient Jurkat cells (Figure S1D, bottom panels), further validating the use of these Abs in IF on isolated cells. To control for staining with anti-pY416 Ab in formalin-fixed samples, two contiguous thin sections of mouse inguinal LNs were treated with or without AP for 30 min at 37º C, respectively, followed by staining with pY416 as in Figure 1D. This experiment showed that AP treatment virtually eliminated IF staining with pY416 (Figure S1E). Furthermore, anti-pY416 staining of thin sections of spleen and splenocytes isolated from part of the same organ showed similar fluorescence intensity (Figure S1G), supporting that the cell isolation procedure did not affect the levels of activated SFKs.

Estimation of the Relative Proportions of the Four Lck Forms

Signal intensity associated to Lck (I_{Lck}) was measured by quantitative immunoblots using near-infrared fluorescence in cell lysates depleted with Lck-phospho-specific or irrelevant Ab (I_{ctr}) (Figure 2). I_{ctr} represented 100% of cellular Lck as irrelevant Ab depleted only trace amounts of Lck with no apparent bias for pY394-Lck or Y394-Lck (Figure S2). After correction for 100% depletion of pY394-Lck or pY505 by anti-pY416 or anti-pY505 (and correction for gel loading), the I_{Lck} / I_{ctr} ratios provided the fraction of Y394-Lck ($\mathbf{F_{Y394}}$) and Y505-Lck ($\mathbf{F_{Y505}}$), respectively. Thus, $\mathbf{F_{pY394}}$ = (1- $\mathbf{F_{Y394}}$) and $\mathbf{F_{pY505}}$ = (1- $\mathbf{F_{Y505}}$). Upon removal of 100% of pY394-Lck and pY505-Lck, a fraction of pY505-Lck and pY394-Lck, respectively, was depleted (Figure 2A, B and Figure 3A, B). This fraction of the pY394-Lck and pY505-Lck pools (readily calculated from the type of experiment shown in Figures 2 A, B and Figure 3A, B) was phosphorylated on both the activation and inhibitory sites and gave similar values ($\mathbf{F_{A/I}}$) with anti-pY416 and anti-pY505 Abs. $\mathbf{F_{A/I}}$ was then used to deduce the fraction of total Lck doubly phosphorylated ($\mathbf{F_{Dpho}}$, = $\mathbf{F_{A/I}}$ X $\mathbf{F_{pY394}}$). Consequently, the fraction of pY394-active Lck was $\mathbf{F_{pY394/Y505}}$ **=** $\mathbf{F_{pY394}}$ - $\mathbf{F_{Dpho}}$ and the fraction of Closed-inactive Lck was $\mathbf{F_{Y394/pY505}}$ **=** $\mathbf{F_{pY505}}$ - $\mathbf{F_{Dpho}}$. Finally, the fraction of “primed” Lck was $\mathbf{F_{Y394/Y505}}$ = 1- ($\mathbf{F_{Y394/pY505.}}$ + $\mathbf{F_{pY394}}$) (or 1 - ($\mathbf{F_{Y394/pY505}}$ **+** $\mathbf{F_{pY394/Y505}}$ + $\mathbf{F_{Dpho}}$). These values for the four Lck forms, indicated in percentage in Table 1, are averages of three determinations. These measurements were corroborated by experiments comparing the fractions of Lck that contained pY505-Lck under native and denatured conditions (Figure 3D), confirming the relative proportions of inactive and Dpho-Lck.

Estimation of the Phosphorylation Rate of TCR ITAMs by Active Lck

Mathematical Model

In what follows we aim to determine the rate at which Lck phosphorylates the T cell receptor ITAMs on a single triggered TCR. Since the enzyme (Lck) and the substrate (TCR ITAMs) are both confined to the plasma membrane, we use the theory presented in Laufenburger and Lindermann (Lauffenburger and Linderman, 1993) for the coupling and uncoupling rates of membrane confined molecules. The Lck-TCR coupling rate is given by,

$$k_c = \frac{k_{on}k_+}{k_{on} + k_+}$$

where k_{on} is the bimolecular reaction on-rate (in units of μm^2/s) and k_+ is the diffusion-limited on-rate given by,

$$k_+ = \frac{2\pi D}{\ln(b/s)}$$

where $D = D_{Lck} + D_{TCR}$ is the sum of the Lck and TCR diffusion coefficients, b is the mean distance between Lck molecules, and s is the reaction radius (approximated by the size of a TCR, s=5 nm). The Lck-TCR uncoupling rate is given by,

$$k_u = \frac{k_+}{k_{on} + k_+}\left(k_{off} + k_{cat}\right)$$

where k_{off} is the dissociation rate and k_{cat} is the rate of phosphorylation.

The mean coupling time is $1/k_cL$ (where L is the concentration of active Lck) and the mean uncoupling time is $1/k_u$. Therefore the mean cycle time for a binding and unbinding event is $1/k_cL + 1/k_u$. The reciprocal of the mean cycle time determines the coupling rate between a population of Lck and a single TCR (see for example (Dushek and Coombs, 2008; Wofsy et al., 2001)

,

$$\frac{1}{1/k_cL + 1/k_u} = \frac{k_cLk_u}{k_cL + k_u}$$

We note that a single Lck-TCR coupling may result in multiple Lck-TCR binding events. This is reflected in the fact that the uncoupling rate k_u depends not only on k_{off} and k_{cat} but also on the escape probability $\gamma = k_+/(k_{on} + k_+)$ (Lauffenburger and Linderman, 1993). In other words, after chemically dissociating the molecules may rebind before diffusing apart. The number of localized binding events is simply

$$\frac{1/k_u}{1/(k_{off} + k_{cat})}$$

We can now calculate the number of Lck-TCR binding events (or `hits') per unit time as,

$$hits/s = \frac{k_cLk_u}{k_cL + k_u} \times \frac{1/k_u}{1/(k_{off} + k_{cat})}$$

As with all enzymatic reactions, only a fraction of hits will lead to phosphorylation and therefore the rate of TCR phosphorylation (k_{phos}) is given by hits/s X k_{cat} /(k_{cat} + k_{off}). After some simplification we find that the rate of TCR phosphorylation by Lck is,

$$k_{phos} = \frac{k_c L}{k_c L + k_{off} + k_{cat}} k_{cat}$$

Parameter Estimation. In order to determine the phosphorylation rate we need to estimate four parameters: k_{on}, k_{off}, k_{cat}, and the concentration of active Lck (L). The catalytic activity of Lck has been previously measured using synthetic peptide substrates to be $k_{cat} = 2\ s^{-1}$ (Watts et al., 1992). We take the concentration of active Lck molecules to be $L = 32\ \mu m^{-2}$ using 10,000 active Lck molecules (measured in the present study) for a cell of radius 5 μm.

The reaction kinetics between membrane molecules are difficult to determine and to our knowledge there is no estimate of these rates between Lck and the TCR ITAMs when both molecules are confined to the plasma membrane. Given that many membrane reactions are diffusion-limited, we expect that $k_{on} > k_+$ and assume that $k_{on}=5k_+$. We estimate that $k_+ \approx 1\ \mu m^2/s$ using reasonable parameters ($D_{Lck} = 0.5\ \mu m^2/s$ (Douglass and Vale, 2005), $D_{TCR}= 0.05\ \mu m^2/s$ (Dushek et al., 2008), b = $(1/\pi L)^{1/2}$ = 0.1 μm, and s = 0.01 μm) and therefore $k_{on} \approx 5\ \mu m^2/s$. Enzyme-substrate dissociation constants are often larger than the catalytic rates ($k_{off} >> k_{cat}$) and a reasonable estimate is $k_{off} = 30\ s^{-1}$ (see for example (Altan-Bonnet and Germain, 2005).

TCR ITAM phosphorylation rate by Lck. Assuming that ITAM phosphorylation is sequential and distributive we can estimate the time required to phosphorylate n ITAMs as $\tau = 2n/k_{phos}$ since each ITAM contains two tyrosine residues. We find that for 10,000 active Lck molecules the time required to phosphorylate n=3 ITAMs (on average) is approximately 3 seconds. In the case of 1000-100 active Lck molecules we find this time to be $\approx$8.5-60 seconds. We observe that when 10,000 molecules of active Lck are present, phosphorylation is limited only by the catalytic rate of the enzyme since the term $k_c L$ is much larger than both k_{off} and k_{cat} in the denominator of k_{phos} (see above). In this calculation we have assumed that TCR do not compete for active Lck, which is expected when few TCR are triggered by low concentration of pMHC. We have assumed distributive and sequential phosphorylation but note that random and/or processive phosphorylation will decrease the time required to phosphorylate TCR ITAMS.

Nika *et al.*

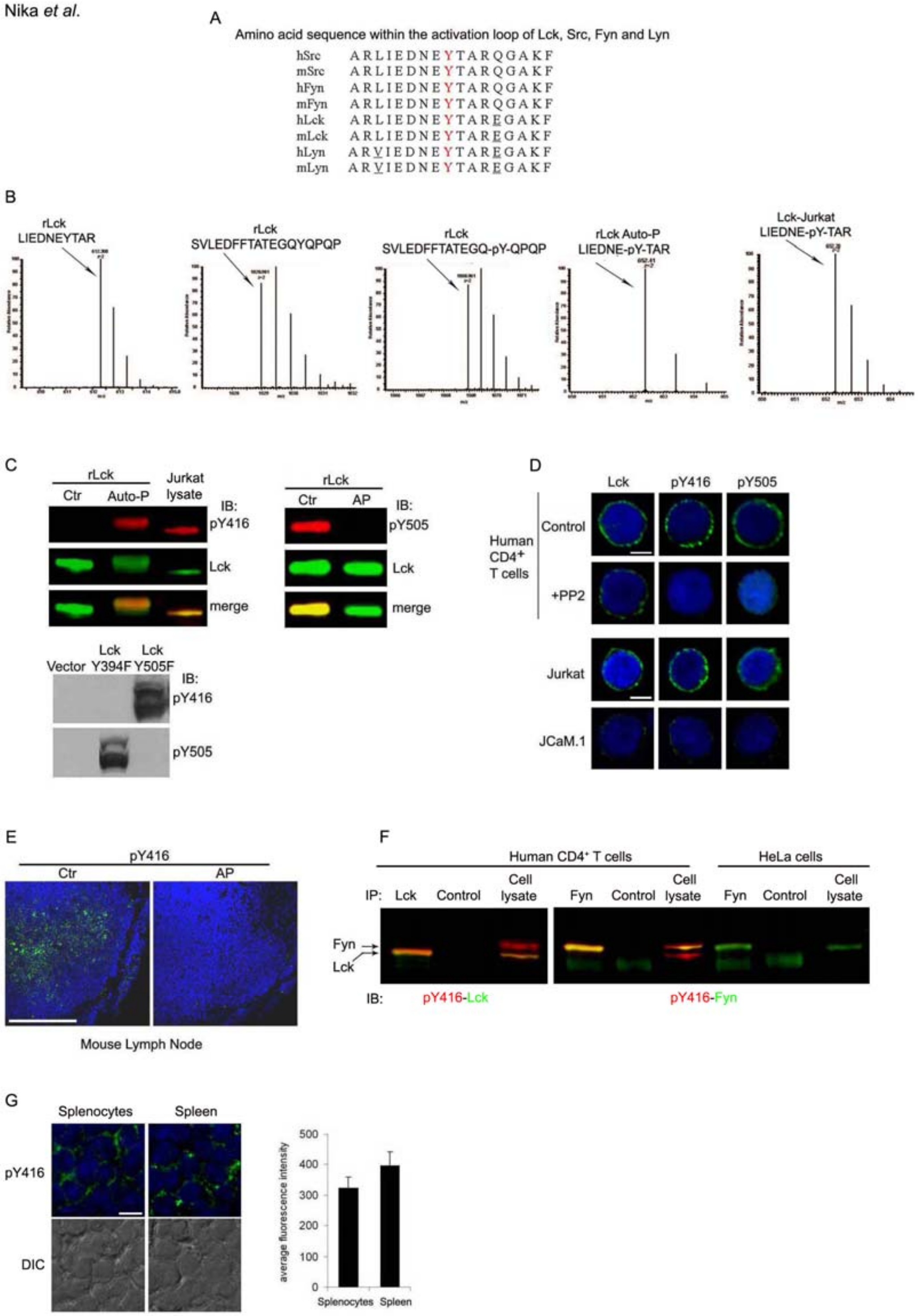

Figure S1, related to Figure 1; Assessment of anti-pY416 and anti-pY505 Abs specificity.

A. Amino acid sequence of the activation loop of human and mouse Src, Fyn, Lck and Lyn. The tyrosine phosphorylated in the activation loop (Y416-Src, Y417-Fyn, Y394-Lck, Y397-Lyn) is shown in red. The sequences are highly conserved, apart from a glutamine to glutamic acid and a leucine to valine substitution (underlined). **B**. MS analysis of rLck and Lck from Jurkat cells. rLck was incubated with or without ATP (Auto-P) for 5 min at 37°C in kinase buffer assay. The reaction mixture was stopped by boiling in Laemmli sample buffer and separated by SDS-PAGE. The gel was stained with colloidal coomassie blue and the band corresponding to rLck was cut and subjected to digestion with trypsin and analyzed by LC-MS-MS (see, Supplemental experimental procedures). Lck was isolated from Jurkat cells (20x10^{6}) with anti-Lck mAb (3A5), separated by SDS-PAGE and processed as above. Arrows indicate the m/z corresponding to relevant peptides analyzed from rLck and Lck from Jurkat. **C**. (top, left-hand panels) Aliquots of the reactions used in **B** (± ATP) were analyzed by immunoblot in parallel with a lysate from Jurkat cells (5x10^{5}) using anti-pY416 Ab (revealed by IRDye680 goat anti-rabbit IgG, pseudo-colour red) and anti-Lck Ab (IRDye800CW goat anti-mouse IgG, pseudo-colour green). Immunoblots were scanned for near-infrared fluorescence in an Odyssey imaging system using 700nm and 800nm channels (see, Supplemental experimental procedures for further details). Overlapping fluorescence (merge) directly reveals the identity of phosphorylated species. (top, right-hand panels) rLck was left untreated (Ctr) or dephosphorylated by AP for 30 min at 37° C. The reaction was separated by SDS-gel and analyzed by immunoblot for pY505 and Lck. (bottom panels) Lck-deficient JCaM.1 cells were transiently transfected by electroporation with empty vector or mutant Y394F-Lck- or Y505F-Lck-containing vector. Cell lysates were separated on SDS-gel and probed with anti-pY416 and anti-pY505 Abs and detected by chemiluminescence. **D**. (Top-two rows) Human CD4^{+} T cells were either left untreated (Control) or incubated with 100μM PP2 (+PP2) for 30 min at 37°C, PFA-fixed and stained with anti-Lck, anti-pY416 or anti-pY505-Lck rabbit Abs followed by Alexa488-secondary Ab. Nuclei were counterstained with Hoechst 33258 (blue) and cells analyzed by confocal microscopy. Scale bar, 3.5μm. (Bottom-two rows) Jurkat cells or JCaM.1 cells were stained with anti-Lck, anti-pY416 or anti-pY505 and analyzed as in the top-two rows. Scale bar, 3.5μm. **E**. Two consecutive 10μm sections of snap frozen mouse LN were formalin-fixed. One was left untreated (Ctr) and the second one incubated with 60U AP for 30min at 37°C and stained with anti-pY416 (green). Nuclei (blue) were counterstained as in **D**. Scale bar, 100μm. **F**. Expression of active SFKs in T cells but not in Hela cells. Lck (left-hand panel), or Fyn (right-hand panel) were

immunoprecipitated from human naïve CD4$^+$ T cells and subjected to dual fluorescence immunoblot analysis with the corresponding Abs (green) and anti-pY416 (red). Coincident fluorescence (yellow) identifies active SFKs. HeLa cell lysate was immunoprecipitated with anti-Fyn (lanes 4-6 of the right-hand panel) and analyzed as above with anti-Fyn (green) and anti-pY416 (red).

G. Confocal IF images of mouse spleen and splenocyte pellet OCT-embedded, snap frozen, formalin-fixed and stained with anti-pY416 (green). (DIC) differential interference contrast image. Histogram shows average fluorescence intensity ±SE (323.7±36 and 396.9±45 n=20 ROI. *P*=0.2399). Scale bar, 5µm.

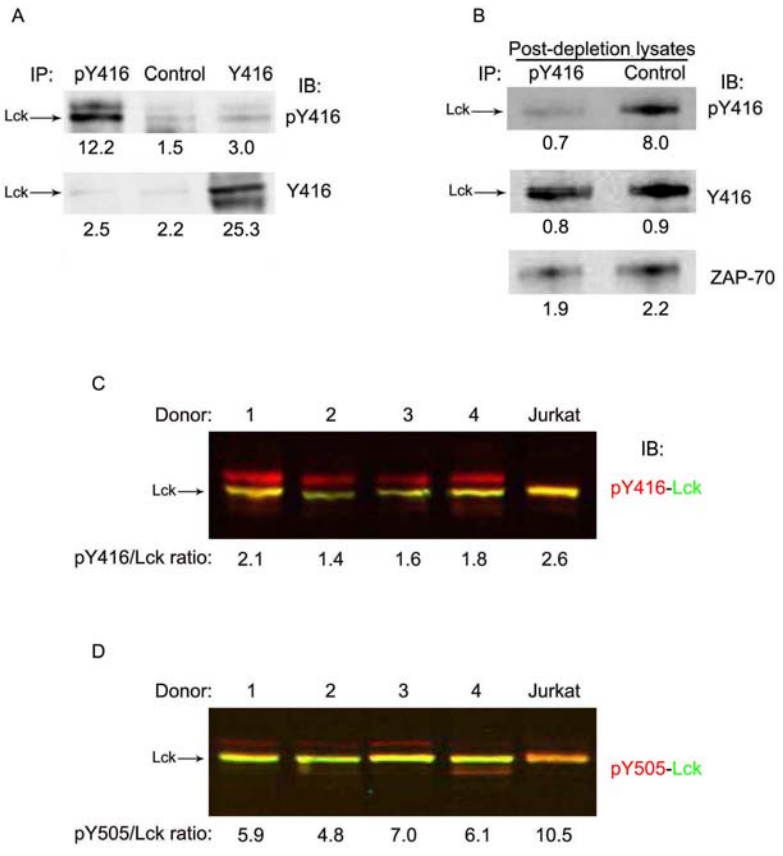

Figure S2, related to Figures 2 and 3.

A and **B.** Minimal cross-reactivity of anti-pY416 and anti-Y416 Abs in immunodepletions**. A**. Denatured Jurkat lysates were divided into three portions and each one subjected to three rounds of immunoprecipitations (IP) with anti-pY416, anti-Y416 Abs (that recognizes non-phosphorylated activation loop of SFKs) or control IgG. Immunoprecipitates were pooled and an aliquot analyzed by dual staining immunoblot with anti-pY416 rabbit Ab or anti-Y416 mAb detected by two-colour

fluorescence and bands quantified (number under the blots). After subtraction of IgG controls, anti-pY416 IP contained ~1% of total inactive Y416-Lck and anti-Y416 IP contained ~12% of pY394-Lck.

B. Aliquots of Jurkat lysates immunodepleted as in **A**, by anti-pY416 or control IgG, were analyzed by immunoblot with anti-pY416 and anti-Y416 and with anti-ZAP-70 as a loading control. Quantification was as in **A**. These controls confirmed that the pool of Y394-Lck minimally contaminated anti-pY416 depletions. However, a slight cross-reactivity of anti-Y416 towards pY394-Lck was detected. Note that Lck phosphorylation status did not change during the entire procedure as pY416 and pY505 remained comparable before and after control rabbit IgG immunodepletion (not shown).

C and **D.** Quantitative immunoblots of pY394-Lck and pY505-Lck content in Jurkat and human naïve $CD4^+$ T cells. Cell lysates of naïve $CD4^+$ T cells isolated from four donors and Jurkat cells were analyzed by dual-colour infrared immunoblot with anti-pY416 (red) and anti-Lck (green) (**A**) or anti-pY505 (red) and anti-Lck (green) (**B**). The ratios of fluorescence signal intensity detected with phospho-specific and Lck Abs, respectively, (pY416/Lck and pY505-Lck/Lck) are indicated. These ratios provide a measure of the relative abundance of pY394-Lck (**A**) or pY505-Lck (**B**) in T cells and Jurkat cells. Knowing the proportion of an Lck form in Jurkat cells, we calculated by proportionality the level of the same form in human T cells (see Results).

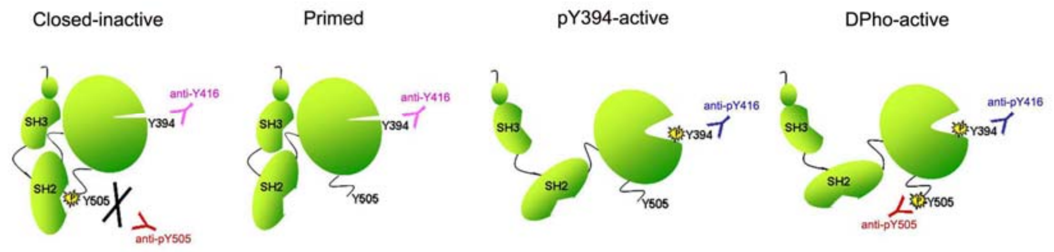

Figure S3, related to Figures 3D and 4; Schematic representation of antibody accessibility to Lck forms

Anti-Y416 (magenta) reacts with Lck closed/inactive and primed forms whereas anti-pY416 (blue) recognizes pY394-active and DPho forms. Anti-pY505-Lck (red) reacts only with native DPho form and not with closed-inactive Lck form but does react with denatured Lck in which the intramolecular interaction between pY505 and the SH2 domain is abolished.

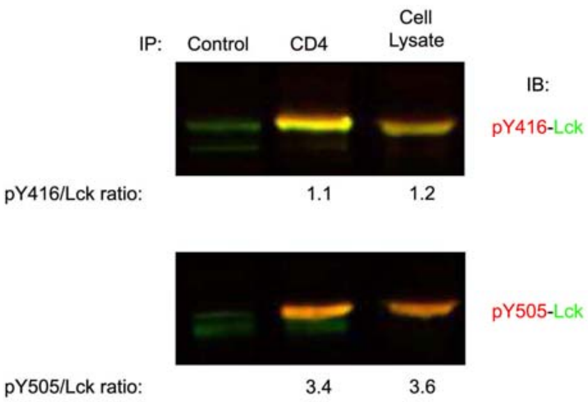

Figure S4, related to Figures 1 and 5; No preferential association of Lck forms with CD4

anti-CD4 or control immunoprecipitations from Jurkat cells were analyzed by dual fluorescence immunoblot with anti-pY416 or anti-pY505 (red) and anti-Lck (green) and bands were quantified. The ratios of fluorescence signal intensity detected with phospho-specific and Lck Abs (pY416/Lck and pY505-Lck/Lck respectively) are indicated. These ratios provide a measure of the relative abundance of pY394-Lck (upper panel) or pY505-Lck (lower panel) contained in the CD4 IP and cell lysate. No preferential association of phospho-Lck species with the co-receptor was observed.

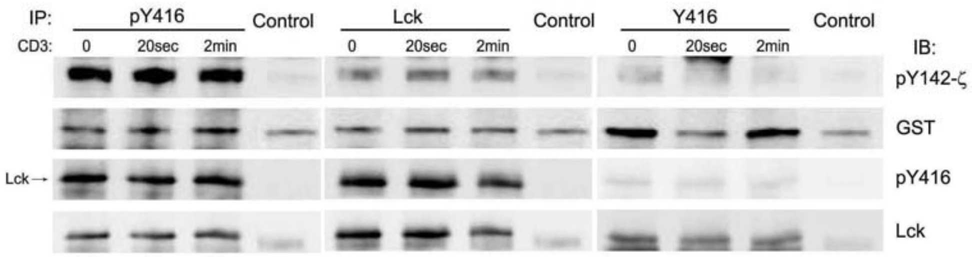

Figure S5, related to Figure 6; Kinase activity of Lck remains unaltered after T cell stimulation

Jurkat cells were stimulated for the indicated times with anti-CD3 mAb. Cell lysates, tested for induction of tyrosine phosphorylation by anti-pY immunoblot (not shown), were immunoprecipitated (IP) with anti-pY416 (left-hand panels), anti-Lck (middle panels) or anti-Y416 (right-hand panels). IPs were used for kinase assays on rCD3ζ–GST and pY142-ζ, GST, pY394-Lck (anti-pY416) and Lck were visualized by immunoblot (IB).

Supplemental Bibliography

Altan-Bonnet, G., and Germain, R.N. (2005). Modeling T cell antigen discrimination based on feedback control of digital ERK responses. PLoS Biol *3*, e356.

Douglass, A.D., and Vale, R.D. (2005). Single-molecule microscopy reveals plasma membrane microdomains created by protein-protein networks that exclude or trap signaling molecules in T cells. Cell *121*, 937-950.

Dushek, O., and Coombs, D. (2008). Analysis of serial engagement and peptide-MHC transport in T cell receptor microclusters. Biophys J *94*, 3447-3460.

Dushek, O., Mueller, S., Soubies, S., Depoil, D., Caramalho, I., Coombs, D., and Valitutti, S. (2008). Effects of intracellular calcium and actin cytoskeleton on TCR mobility measured by fluorescence recovery. PLoS One *3*, e3913.

Gerber, S.A., Rush, J., Stemman, O., Kirschner, M.W., and Gygi, S.P. (2003). Absolute quantification of proteins and phosphoproteins from cell lysates by tandem MS. Proc Natl Acad Sci U S A *100*, 6940-6945.

Lauffenburger, D.A., and Linderman, J. (1993). Receptors: Models for Binding, Trafficking and Signaling. . Oxford University Press.

Marvizon, J.C., Perez, O.A., Song, B., Chen, W., Bunnett, N.W., Grady, E.F., and Todd, A.J. (2007). Calcitonin receptor-like receptor and receptor activity modifying protein 1 in the rat dorsal horn: localization in glutamatergic presynaptic terminals containing opioids and adrenergic alpha2C receptors. Neuroscience *148*, 250-265.

Rappsilber, J., Ishihama, Y., and Mann, M. (2003). Stop and go extraction tips for matrix-assisted laser desorption/ionization, nanoelectrospray, and LC/MS sample pretreatment in proteomics. Anal Chem *75*, 663-670.

Shevchenko, A., Tomas, H., Havlis, J., Olsen, J.V., and Mann, M. (2006). In-gel digestion for mass spectrometric characterization of proteins and proteomes. Nat Protoc *1*, 2856-2860.

Watts, J.D., Wilson, G.M., Ettenhadieh, E., Clark-Lewis, I., Kubanek, C.A., Astell, C.R., Marth, J.D., and Aebersold, R. (1992). Purification and initial characterization of the lymphocyte-specific protein-tyrosyl kinase p56lck from a baculovirus expression system. J Biol Chem *267*, 901-907.

Wofsy, C., Coombs, D., and Goldstein, B. (2001). Calculations show substantial serial engagement of T cell receptors. Biophys J *80*, 606-612.

Woods, A.S., and Ferre, S. (2005). Amazing stabililty of the arginine-phosphate electrostatic interaction. J Proteome Res 4, 1397-1402.
